# Role of long noncoding RNAs; BDNF-AS and 17A and their relation to GABAergic dysfunction in Egyptian epileptic patients

**DOI:** 10.1007/s11011-023-01182-x

**Published:** 2023-02-20

**Authors:** Aya A. Zayed, Mae M. Seleem, Hebatallah A. Darwish, Amira A. Shaheen

**Affiliations:** 1 Department of Pharmacology, Toxicology and Biochemistry, Faculty of Pharmacy, Future University in Egypt, Cairo, Egypt; 2 Department of Biochemistry, Faculty of Pharmacy, Cairo University, Cairo, Egypt

**Keywords:** Idiopathic and symptomatic epilepsy, lncRNA BDNF-AS, lncRNA 17A, BDNF, CREB, Glutamate/ GABA ratio

## Abstract

Epilepsy is a chronic neurological disorder characterized by recurrent unprovoked seizures. Lately, long noncoding RNAs (lncRNAs) have been increasingly appreciated as regulators of epilepsy-related processes, however, their functional role in its pathogenesis is still to be explored. This study investigated the expression levels of lncRNAs; BDNF-AS and 17A in the sera of Egyptian patients with idiopathic generalized and symptomatic focal epilepsy and correlated their levels with brain-derived neurotrophic factor (BDNF), phosphorylated cAMP reaction element -binding protein (p-CREB), gamma- aminobutyric acid (GABA) and glutamate, to underline their related molecular mechanism. A total of 70 epileptic patients were divided into two clinical types, besides 30 healthy controls of matched age and sex. The expression levels of both lncRNAs were markedly upregulated in epileptic groups versus the healthy control group with predominance in the symptomatic focal one. Epileptic patients showed significantly lower levels of BDNF, p-CREB, GABA along with significant increase of glutamate levels and glutamate/ GABA ratio, especially in symptomatic focal versus idiopathic generalized epileptic ones. The obtained data raised the possibility that these lncRNAs might be involved in the pathogenesis of epilepsy via inhibition of GABA/p-CREB/BDNF pathway. The study shed light on the putative role of these lncRNAs in better diagnosis of epilepsy, particularly symptomatic focal epilepsy.

## Introduction

Epilepsy is the fourth most common neurological disorder affecting people of all ages with prevalence of about 70 million globally (Sopic et al. 2018). It is a chronic non-communicable disease of the brain, characterised by the presence of recurrent and unprovoked seizures as common features of the epileptic condition (Pákozdy et al. 2008). These seizures are episodes that vary from brief and nearly undetectable periods to long periods of vigorous shaking, resulting in unusual behaviour and sometimes loss of awareness and physical injuries (Devinsky 2007). The etiology of epileptic seizures is multi-factorial, where the onset, susceptibility and progression are influenced by a range of genetics and acquired factors or their interaction in many cases (Sirven 2015). Etiological classification of epilepsy includes four categories: idiopathic, symptomatic, provoked, and cryptogenic (Berg et al. 2010; Shorvon 2011). Herein, we focused on idiopathic and symptomatic ones. Idiopathic epilepsy is predominately of genetic origin with no gross neuroanatomical or neuropathological abnormality, whereas, the symptomatic is associated with gross anatomical or pathological anomalies and/or clinical features that are reflective of an underlying disease or condition (Shorvon 2011). To date, biomarkers that might provide objective criteria to confirm or rule out diagnosis of epilepsy are lacking, making epilepsy difficult to diagnose since a number of other conditions may share very similar signs and symptoms of seizures such as syncope, migraine, narcolepsy, pain attacks and others (Crompton and Berkovic 2009). Hence, understanding the molecular mechanism of epilepsy pathogenesis would aid in the early detection and the optimal disease management.

In epileptic seizures, the principal mechanistic scenario is viewed as a shift in balance between the inhibitory gamma-aminobutyric acid (GABA) and the excitatory glutamate neurotransmission in favour of the latter (Treiman 2001). This shift occurs due to selective loss of inhibitory GABA-ergic neurons after precipitating epileptogenic insult as well as the reorganization of neuronal circuits, resulting in aberrant hypersynchronous electrical activity of neuronal network (Villa et al. 2019). Generated GABA in GABA-ergic axon terminal with its release in the synapse, mediates its action via one of two receptors; GABA_A_ which regulates chloride entry and GABA _B_ which increases potassium conductance, decreases calcium entry, and prevents the presynaptic release of other transmitters. GABA retains the inhibitory tone to balance neuronal excitation which if disrupted, can lead to seizures (Treiman 2001). Deficit in GABA mediated signalling and augmentation of glutamatergic transmission have been documented in many types of epilepsy (Czuczwar and Patsalos 2001; Landmark 2008; Lasoń et al. 2013). Moreover, this deficit has been linked to a decrease of cAMP response element-binding protein (CREB) phosphorylation and activation (Jagasia et al. 2009). Studies have indicated that a variety of CREB target genes are closely related to epilepsy including: α_1_ subunit of GABA_A_ receptor, brain-derived neurotrophic factor (BDNF), cyclooxygenase 2 (COX-2) and N-methyl D-aspartate receptor subtype 2B (NR2B) (Wang et al. 2020). Previously, Hu et al. (2008) demonstrated that CREB over expression significantly suppresses the activity of α_1_ GABA_A_, indicating a critical role of CREB in the regulation of α_1_ subunit-expression. Likewise, BDNF, a potent pro-survival factor, is involved in synaptic plasticity, learning, and memory processes (Yamada et al. 2002; Gómez-Palacio-Schjetnan and Escobar 2013). Mohammadi et al. (2018) reported that the neuroprotective impact of BDNF is linked to CREB signalling activity in neurodegenerative diseases. Moreover, a positive feedback loop showing a mutual interaction between BDNF and CREB has been demonstrated in animal models of epilepsy (Wang et al. 2020). Nevertheless, molecular mechanism responsible for epileptogenesis are still under investigation.

Long non-coding RNAs (lncRNAs), a class of long transcripts without protein-coding capacity, have emerged as regulatory molecules that are implicated in wide variety of biological processes. Lately, they are increasingly appreciated for their involvement in regulation of epilepsy-related molecular processes, particularly those which are already associated with brain excitability and seizures (Henshall and Kobow 2015). However, the exact role and the impact of lncRNAs in epileptogenesis is beginning to be explored and further research need to be done.

Brain-derived neurotrophic factor antisense RNA (BDNF-AS) was the first lncRNA addressed in the current study. It is a lncRNA produced from the opposite strand of BDNF. A potential role for BDNF-AS in post transcription regulation of BDNF has been suggested, since BDNF and BDNF-AS form double-stranded duplexes (Marler et al. 2014). *In vitro* cell line studies showed that BDNF-AS can negatively regulate BDNF expression in PC12 and BV-2 cells (Jang et al. 2018; Zhang et al. 2018; Zhi and Lian 2019). Whereas, in an epilepsy study, the expression of BDNF was upregulated in human cortex removed as surgical treatment together with concomitant decrease in BDNF-AS expression (Lipovich et al. 2012). These data suggest a sort of interaction of mRNA-lncRNA that might represent a regulatory network of human brain plasticity in which inhibition of BDNF signalling could be used as a potential strategy for epilepsy management.

LncRNA 17 A is another interesting ncRNA involved in neurodegenerative disorder such as Alzheimer disease (AD) (Massone et al. 2011). 17 A was identified as polymerase III-dependent ncRNA that is embedded in the intron 3 of human G-protein-coupled receptor 51 gene (GPR51 that codes for GABA_B2_ receptor; GABA_B_ R_2_). In SH-SY_5_Y cell lines as an *In vitro* model for AD, Massone et al. (2011) demonstrated an alternative splicing of GPR51 gene under influence of 17A, generating a different transcript isoform that abolishes GABA_B__2_ intracellular signalling and impairs its transductional activity. Accordingly, we anticipate that such observation is likely to occur in other neurologic disorder such as epilepsy.

There is a possibility that epileptogenesis might be linked to a variety of changes in gene expression and function of neuro-mediators such as BDNF, CREB, GABA and glutamate, all of which contribute to deregulated neuronal circuits with a proclivity for synchronous electrical activity (Hewson et al. 2016). Since, up till now, there are no data available concerning the involvement and significance of lncRNAs; BDNF-AS and 17A as biomarkers in epilepsy, the present study was directed to investigate their serum expression levels in Egyptian patients with idiopathic generalized and symptomatic focal epilepsy. It also aimed to identify the molecular mechanism implicated in epilepsy by correlating the levels of these neuro-mediators with each other as well as the studied lncRNAs, respectively.

## Subjects & methods

### Study participants

The study included a total of 100 Egyptian participants; divided into 30 healthy controls and 70 epileptic patients of matched age and sex, recruited from the Neurology Clinics, Neurology Departments; Al Kasr Al-Ainy Hospital, Cairo University and Al Demrdash hospital, Ain Shams University, from June 2019 to February 2020 and from September 2020 to November 2020, respectively. Full general and neurological examinations were conducted by a supervising neurologist following Scottish Intercollegiate Guidelines Network 2018 (SIGN 143; Diagnosis and management of epilepsy in adults). Epileptic patients were further classified into two groups: 35 suffering from idiopathic generalized epilepsy and 35 with focal symptomatic epilepsy. Eligible patients were those above 18 years old and diagnosed with epilepsy according to the EEG provided by treating physician. The exclusion criteria included any current or recent inflammatory or infectious disease detected during the previous month, pregnancy, diabetes, those suffering from malignancy and other neurodegenerative diseases.

### Blood sampling

Five millilitres of venous blood samples were collected from all participants using serum collection tubes. The separated sera were aliquoted and stored at − 80 °C for subsequent determination of the expression levels of lncRNAs; BDNF-AS and 17A as well as the levels of BDNF, p-CREB, GABA and glutamate.

## Methodology

### I. Serum lncRNAs assay using quantitative real‑time polymerase chain reaction (qRT-PCR)

Total RNA was extracted from 250 μL serum samples using the miRNeasy Mini Kit (Qiagen, Germany) according to the manufacturer's instructions (Qiagen, Germany). The resultant RNA was then dissolved in 50 μL of RNase-free water and stored at − 80 °C till analysis. RNA quality was determined using nanodrop (Thermo scientific, USA) where the purity range for samples was 1.8–2.0 at wavelength 260/280 with RNA yield ranging from 700 to1400 ng. Reverse transcription (RT) was done using RT2 first strand Kit (Qiagen, Hilden, Germany) where 11 μL total RNA template were reversely transcribed in a final reaction mix volume of 20 μL. For the synthesis of cDNA, the RT reaction was incubated for 60 min at 37 °C and for 5 min at 95 °C. The cDNA produced was stored at − 20 °C till analysis.

Relative expression levels of the lncRNAs; BDNF-AS and 17A were evaluated using the RT2 SYBR Green Master Mix kit (Qiagen, Hilden, Germany) according to the manufacturer's instructions. The housekeeping gene, glyceraldehyde-3-phosphate dehydrogenase (GAPDH), was selected as the internal control. Briefly, 5 μL cDNA product was used as a template in a 25 μL total reaction volume containing 12.5 μL RT2 SYBR Green PCR Master Mix, 6.5 μL nuclease-free water and 1 μL RT2 lncRNA PCR primer assay. Readily made primers by Qiagen were used for amplification. The primer sequences were provided in Table 1. qRT-PCR was performed with a Qiagen Rotor Gene Q6 Plex Real-Time PCR system (Qiagen, Hilden, Germany), with a PCR initial activation at 95 °C for 10 min, followed by 40 cycles of 95 °C for 15 s and 60 °C for 60 s. Data were analysed with Rotor Gene Q software with the automatic threshold cycle (Ct) setting. The relative expression for each lncRNA after normalization to GAPDH was calculated using the 2^−ΔΔct^ method.

**Table 1:** Primer sequences used for real time PCR

Gene	Sequences
lncRNA BDNF-AS	Forward 5`TACCACAAGGTACCAACCATATATG 3` Reverse 5`CATGTGGTTCTGTTTCAATGCCC 3`
lncRNA 17-A	Forward 5` CCACCCTGCAACTGACACAT 3` Reverse 5` GCAAAGGTGCTAATCTTGACTCTTG 3`
GAPDH	Forward 5′GGAGCGAGATCCCTCCAAAAT-3′ Reverse 5′-GCTGTTGTCATACTTCTCATGGA-3′

BDNF-AS: Brain-derived neurotrophic factor antisense RNA, 17A: RNA polymerase (pol) III-dependent, GAPDH: Glyceraldehyde 3-phosphate dehydrogenase

### II Serum levels of BDNF, p-CREB, GABA and glutamate

Serum levels of BDNF, p- CREB, GABA and glutamate were assayed using ELISA kits (DuoSet® IC ELISA, Catalog Number: DYC2510-2), (Cloud-Clone Corp, Catalog Number: SCA011Hu), (My BioSource CA USA, Catalog Number. MBS9337182) and (Novus Biologicals USA, Catalog Number: KA1909), respectively according to the manufacturer's instructions and expressed as pg/mL for BDNF and p- CREB and as ng/ml for GABA and glutamate.

### Statistical analysis

The results were presented as mean ± standard error of mean (M ± SEM). Both parametric and nonparametric statistical methods were used to give full study of different types of epilepsy. Power analysis was conducted for one-way fixed-effect analysis of variance (ANOVA) to compare between different groups followed by post Hoc Tukey multiple comparison test. Nonparametric receiver operating characteristic (ROC) curves were created between idiopathic generalized and symptomatic focal groups in which the value for sensitivity is plotted against 1-specifcity. A prognostic test (positive versus negative) was conducted for idiopathic generalized and symptomatic focal groups using a cut-off threshold for lncRNA BDNF-AS, lncRNA 17 A, BDNF, p-CREB, GABA and glutamate. The positivity rates were compared by chi-square test. The overall accuracy of a molecular marker to predict different types of epilepsy is defined as the average of the sensitivity and the specificity. Simple linear regression analysis was applied between idiopathic generalized and symptomatic focal patients using Pearson χ2 test to study the correlation of serum levels of lncRNAs; BDNF-AS and 17A with demographic and clinical data, BDNF, p-CREB, GABA and glutamate as well as their ratio. All statistical analyses were performed using Windows-based SPSS statistical software (SPSS version 20.0, SPSS Inc., Chicago, IL) and GraphPad Prism 7.0 (GraphPad Software, CA, USA) using IBM SPSS Statistics 20 program.

## Results

### Demographic and clinical characteristics of the study participants

Table 2 provides the demographic and clinical characteristics of the study participants. As shown, there are 30 healthy individuals and 70 epileptic patients; of them, 35 were idiopathic generalized and 35 were symptomatic focal epileptic patients with disease onset equivalent to 16.25 ± 1.26 and 18.5 ± 2.2 years and mean duration of disease of 17.74 ± 2.03 and 19.78 ± 1.88 years, respectively. Moreover, gender and age were relatively matched among the study groups.

**Table 2:** Demographic and clinical characteristics of studied groups

Groups	Control *N* = 30	Idiopathic generalized *N* = 35	Symptomatic focal *N* = 35	*P* value
Parameters				
*Gender:*				0.017
Male	18 (60%)	26 (74.2%)	22 (62.8%)	
Female	12 (40%)	9 (25.8%)	13 (37.2%)	
*Age (Y)* (Median) range	31.5 (19–65)	35 (19–53)	37 (19–72)	0.256
*Disease onset (Y)*		16.25 ± 1.26	18.5 ± 2.2	0.430
*Duration (Y)*		17.74 ± 2.03	19.78 ± 1.88	0.464
*Etiology*				0.000
• 2ry structure		0 (0%)	32 (91.4%)	
• 2ry structure with systemic lupus erythematosus		0 (0%)	3 (8.6%)	
• Unknown		32 (91.4%)	0 (0%)	
• Genetics		3 (8.6%)	0 (0%)	
*Types of seizures*				
Generalized seizures:				0.034
• Tonic- Clonic (Grand mal)		1 (2.8%)	3 (5.7%)	
• Absence seizures (Petit mal)		1 (2.8%)	0 (0%)	
Focal seizures:				
• Focal evolving to generalized		33 (94.2%)	25 (71.4%)	
• Focal		0 (0%)	7 (20%)	
*Cognitive/affections*				1.000
• Yes		2 (5.7%)	2 (5.7%)	
• No		33 (94.2%)	33 (94.2%)	
*Neurological examination*				0.146
• Nocturnal /diurnal		0 (0%)	2 (5.7%)	
• Nocturnal		35 (100%)	30 (85.7%)	
• Ophthalmoplegia		0 (0%)	1 (2.8%)	
• Port-wine stain (Struge- Weber syndrome)		0 (0%)	2 (5.7%)	
*EEG*				0.098
• Normal	30 (100%)	33 (94.2%)	29 (82.2%)	
• A focal right temporal sharp wave in the anterior temporal region		0 (0%)	2 (5.7%)	
• Diffuse slowing		2 (5.7%)	2 (5.7%)	
• Right temporal interictal activity and left temporal epileptogenic dysfunction		0 (0%)	2 (5.7%)	

N: number, Y: year, Significant at *p* < 0.05

Regarding the etiology of the disease, the current data revealed that 91.4% of idiopathic generalized patients suffered from structural abnormality due to unknown causes, whereas symptomatic focal patients suffered mostly from 2ry structural abnormality due to tumors (91.4%). Regarding seizure types, 94.2% of idiopathic generalized and 71.4% of symptomatic focal suffered from focal evolved to generalized seizures. However, 94.2% of patients in both groups maintained their cognitive abilities.

Likewise, the neurological examination of patients revealed that 100% of idiopathic generalized patients and 85.7% of symptomatic focal patients exhibited nocturnal attacks. Meanwhile, the EEG examinations showed that 94.2% of idiopathic generalized patients and 82.2% of symptomatic focal patients were normal. Remarkedly, all patients were using their prescribed anti-epileptic drugs (valproic acid, Carbamazepine, Phenytoin and Levetiracetam).

### Serum expression levels of lncRNAs; BDNF-AS and 17A in healthy control and epileptic groups

**Fig. 1:**
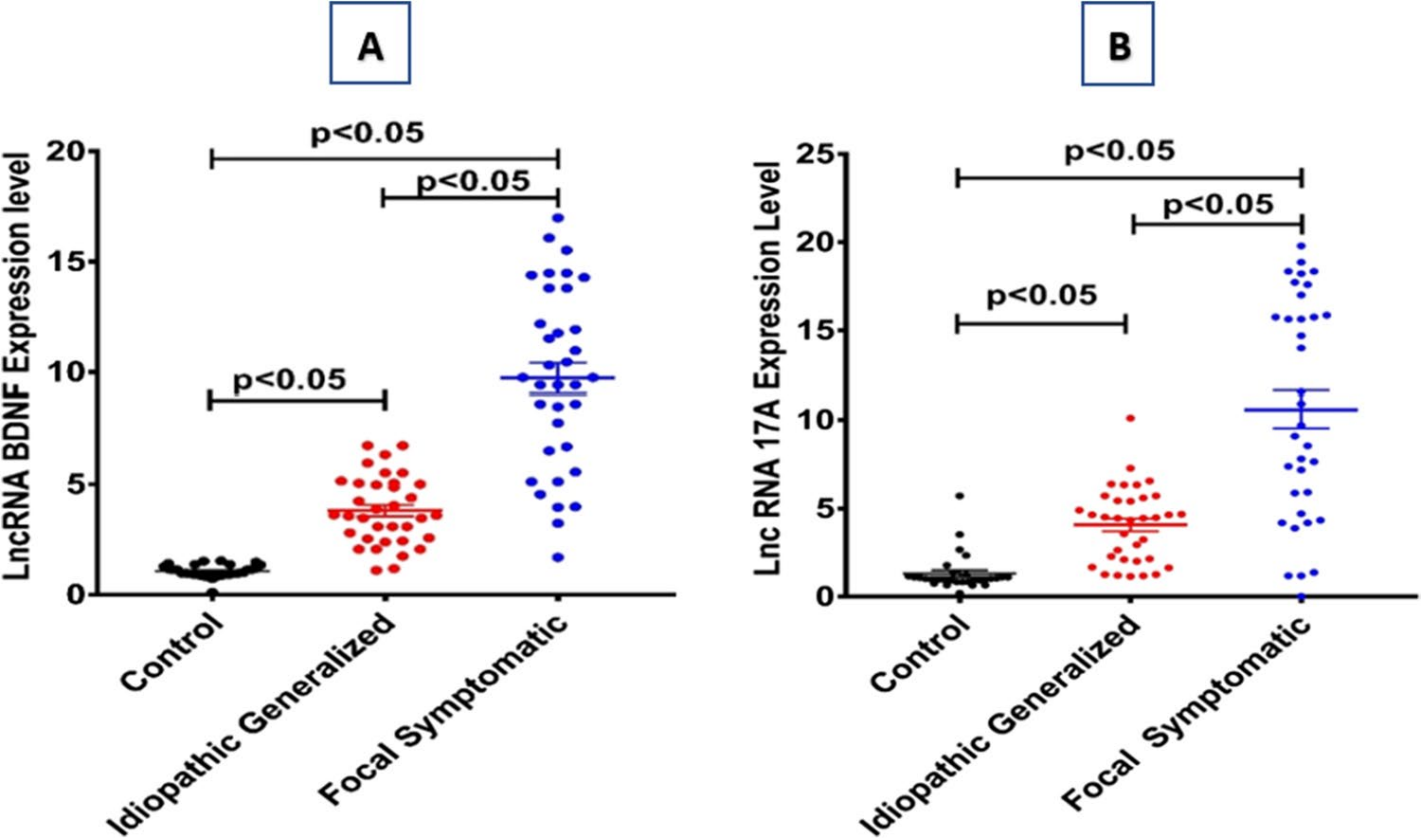
Scatter plot of the distribution of lncRNA BDNF-AS (**A**) and lncRNA 17A (**B**) in the sera of control (*n* = 20), idiopathic generalized (*n* = 35) and symptomatic focal (*n* = 35) epileptic patients. Bars indicate median with interquartile range. All fold changes are relative to the healthy control group. Significant *P*-values are indicated on graph

The expression levels of lncRNAs; BDNF-AS and 17A were markedly upregulated in epileptic groups as compared to healthy control. Moreover, the expression levels of both lncRNAs were **s**ignificantly higher in symptomatic focal patients compared to idiopathic generalized ones at *p* value < 0.05 (Fig. 1).

### Serum levels of BDNF and p-CREB in healthy control and epileptic groups

**Fig. 2:**
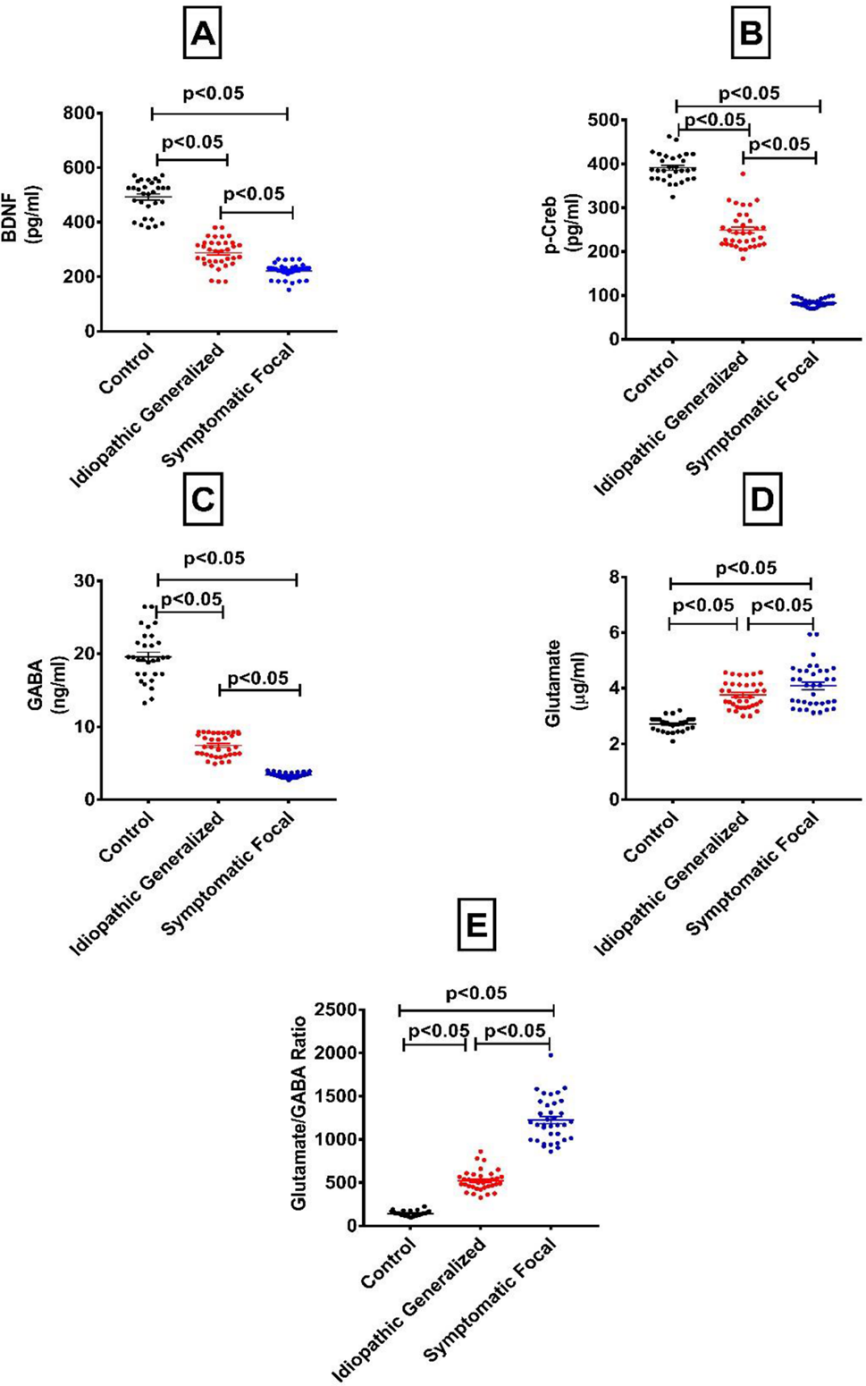
Scatter plot of the distribution of BDNF (**A**), p-CREB (**B**), GABA B (**C**), Glutamate (**D**) and Glutamate/GABA Ratio (**E**) in the sera of control (*n* = 30), idiopathic generalized (*n* = 35) and symptomatic focal (*n* = 35) epileptic patients. Bars indicate median with interquartile range. Significant *P*-values are indicated on graph

As depicted in Fig. 2, the serum levels of BDNF and p-CREB were low in all patients compared to healthy control group, meanwhile, their levels were significantly lower in symptomatic focal group than idiopathic generalized ones at *p* value < 0.05.

### Serum levels of GABA, glutamate, and their ratio

Compared to healthy individuals, epileptic patients showed a significant decrement in GABA levels along with a significant increment in glutamate levels. Meanwhile, the Glutamate/GABA ratio was significantly increased with higher values in symptomatic focal patients compared to idiopathic generalized ones at *p* value < 0.05 (Fig. 2).

### Correlation analysis of different serum molecular markers in epileptic groups

To evaluate the usefulness of lncRNAs; BDNF-AS and 17 A as epilepsy biomarkers, we tested whether their levels were associated with clinical and biochemical data. As shown in table (3), a negative correlation was observed between each of lncRNAs and the etiology.

On the other hand, a significant positive correlation between lncRNAs; BDNF-AS and lncRNA 17A along with negative correlations between each of them and the serum levels of BDNF, p-CREB and GABA were detected. Positive correlation between each of the lncRNAs and Glutamate/GABA ratio was also observed (Table 3). Additionally, significant positive correlations were detected between BDNF, p-CREB and GABA levels with each other, whereas they are negatively correlated with glutamate and glutamate/GABA ratio (Table 4).

**Table 3:** Correlation analysis of serum lncRNA BDNF-AS and lncRNA 17A with clinical and biochemical markers in epileptic patients

	lnc BDNF-AS	lnc 17A
Age	N.S	N.S
Onset	N.S	N.S
Etiology	-0.675**	-0.316**
lnc BDNF AS	–––––	0.558**
lnc 17A	0.558**	–––––
BDNF	-0.671**	-0.494**
p-CREB	-0.778**	-0.573**
GABA	-0.679**	-0.523**
Glutamate	N.S	0.297*
Glutamate/ GABA	0.778**	0.637**

Pearson’s correlation analysis was used to measure the statistical relationship between two variables

Significant correlation at *p* < 0.05* and < 0.001**, respectively. N.S: Non- significant

**Table 4:** Correlation analysis between serum BDNF, p-CREB, GABA and Glutamate as well as their ratio

	BDNF	p-CREB	GABA	Glutamate
BDNF	–––––––	0.818**	0.869**	-.685**
p-CREB	0.818**	–––––––	0.891**	-0.638**
GABA	0.869**	0.891**	–––––––	-0.677**
Glutamate	-0.685**	-0.638**	-0.677**	–––––––
Glutamate/GABA	-0.781**	-0.907**	-0.828**	0.771**

Pearson’s correlation analysis was used to measure the statistical relationship between two variables

Significant correlation at *p* < 0.05* and < 0.001**, respectively. N.S: Non- significant

### ROC curve and positivity rates

**Fig. 3:**
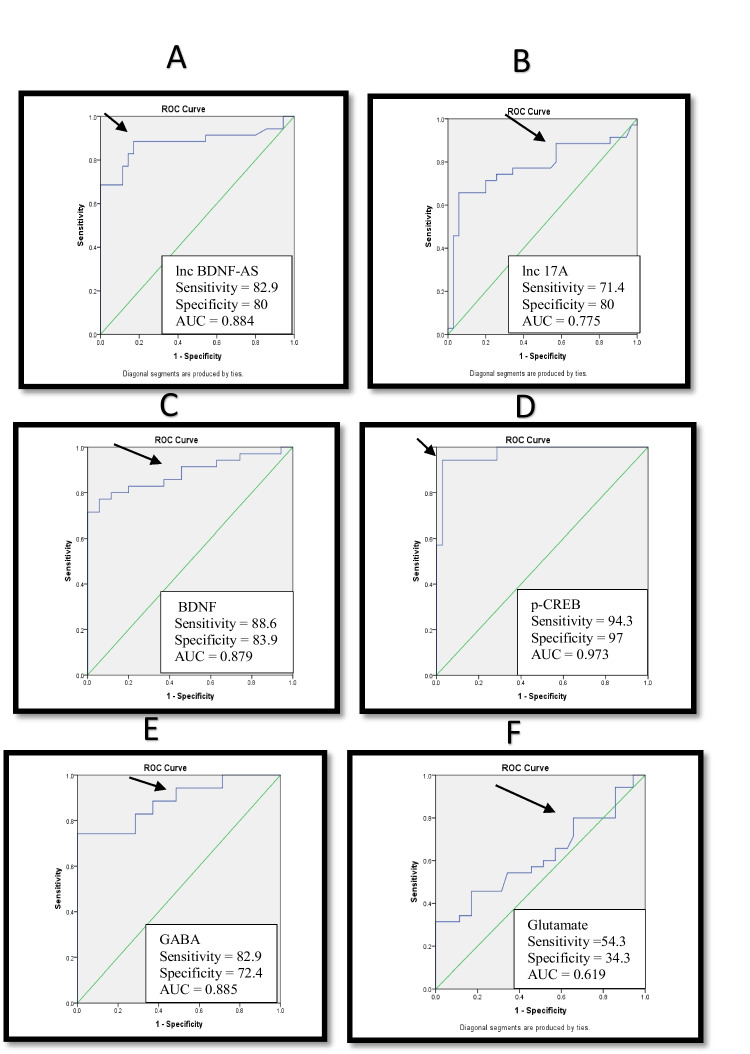
Receiver operator characteristic curve (ROC) of lncRNA BDNF-AS (**A**), lncRNA 17A (**B**), BDNF (**C**), p-CREB (**D**), GABA (**E**) and Glutamate (**F**) in idiopathic generalized and symptomatic focal epileptic patients. The arrows denote the best cutoff point

Receiver operator characteristic curve (ROC) was performed to test the possibility of using lncRNAs; BDNF-AS and 17A as biomarkers in the diagnosis and the differentiation between idiopathic and symptomatic epilepsy. For BDNF-AS, the optimal cut-off value was 5.087-fold change giving 82.9% sensitivity, 80% specificity and area under the curve (AUC) of 0.884. Whereas, for lncRNA 17A, the optimal cut-off was 5.804-fold change giving 71.4% sensitivity, 80% specificity and AUC equivalent to 0.775. Also, p-CREB showed an optimal cut-off of 321.1 pg\ml giving 94.3% sensitivity, 97% specificity and AUC of 0.885. For BDNF, the optimal cut-off value was 238 pg/ml giving 88.7% sensitivity, 83.9% specificity and AUC of 0.879 and for GABA, the optimal cut-off was 13.5 ng\ml giving 82.9% sensitivity, 72.4% specificity and AUC equivalent to 0.885. Finally, the optimal cut-off for glutamate was 4.005 µg/ml giving 54.3% sensitivity, 34.3% specificity and 0.605 AUC (Fig. 3).

Regarding the positivity rates, they were 20% for both lncRNAs in the serum of idiopathic generalized epilepsy, compared to 82.9% for lncRNA BDNF-AS and 71.4% for lncRNA 17A in case of symptomatic focal epilepsy. Moreover, the positivity rates were 11.4% for BDNF, 5.7% for p-CREB, 28.6% for GABA and 34.3% for glutamate in idiopathic generalized group versus 82.9%, 94.3%, 85.7% and 54.3%, respectively in symptomatic focal group. These data pointed to the usefulness of these lncRNAs and the studied neuro-mediators, with the superiority of p- CREB, in differentiating both types of epilepsy in the current study (Table 5).

**Table 5:** Positivity rates of lncRNA BDNF-AS and lncRNA 17A among idiopathic generalized, symptomatic focal, and control groups

Groups	Idiopathic generalized (*n* = 35) %	Symptomatic focal (*n* = 35) %	Control (*n* = 30) %	Chi Square	*P* value
Serum marker					
*lncRNA BDNF-AS*					
No of cases ≥ 5.0867	7 (20%)	29 (82.9%)	0 (0%)	27.680	0.00
*lncRNA 17A*					
No of cases ≥ 5.804	7 (20%)	25 (71.4%)	0 (0%)	18.651	0.00
*BDNF*					
No of cases ≤ 238	4 (11.4%)	29 (82.9%)	0 (0%)	35.831	0.00
*p-CREB*					
No of cases ≤ 321.1	2 (5.7%)	33 (94.3%)	0 (0%)	54.914	0.00
*GABA*					
No of cases ≤ 13.55	10 (28.6%)	30 (85.7%)	0 (0%)	23.33	0.00
*Glutamate*					
No of cases ≥ 4.005	12 (34.3%)	19 (54.3%)	0 (0%)	2.837	0.00

### Univariate logistic regression analysis for predicting symptomatic focal epilepsy

To predict the risk of symptomatic focal epilepsy, univariate logistic regression analysis was conducted. Data indicated that lncRNA BDNF-AS, lncRNA 17 A, BDNF, p-CREB and GABA were significant predictors for symptomatic focal epilepsy with odds ratios: 19.33, 10.00, 0.027, 272.25, and 15.00, respectively. Remarkably, p-CREB showed the highest odds ratio (272.25) (Table 6).

**Table 6:** Univariate logistic regression between idiopathic generalized and focal symptomatic epileptic groups

Parameter	B	S.E	*p*-value	Odd ratio	Odd ratio 95% (CI)
lncRNA BDNF-AS	2.961	0.616	0.000	19.333	5.778-64.689
lncRNA 17A	2.302	0.564	0.000	10.000	3.308-30.230
BDNF	-3.623	0.695	0.000	0.027	0.007-0.104
p-CREB	5.606	1.030	0.000	272.25	36.170-2049.198
GABA	2.708	0.611	0.000	15.000	4.529-49.680

B: Coefficient, S.E: Standard error, C.I: Confidence Interval

## Discussion

Epilepsy is a neurological illness defined by aberrant electrical activity in the CNS, with recurrent seizures as one of the most important symptoms (Villa et al. 2019; Akyuz et al. 2020; Wang 2021). Although, inflammation (Vezzani et al. 2011), alternation in synaptic strength (Leite et al. 2005), apoptosis (Henshall 2007), and ion channel malfunction (Lerche et al. 2013) have been reported as underlying pathogenic processes, the pathophysiology of epilepsy is still not fully understood (Blauwblomme et al. 2014).

LncRNAs have emerged as regulatory molecules involved in a wide variety of biological processes. A rising number of studies revealed that they were dysregulated during epileptogenesis (Shao and Chen 2017; Hashemian et al. 2019; Villa et al. 2019). Interestingly, this current study was the first to investigate the usefulness of serum lncRNAs; BDNF-AS and 17A expression as diagnostic biomarker of epilepsy. It also aimed to emphasize their relation to BDNF, p-CREB, GABA and glutamate as means for further insight into the underlying molecular mechanism.

The present data revealed significant upregulations in the serum expression levels of lncRNAs; BDNF-AS and 17A in the diseased groups as compared to the healthy control, together with significant differences between idiopathic and symptomatic epileptic patients. Similarly, Zhong et al. (2017) found that lncRNA BDNF-AS was considerably upregulated in individuals with cerebral infarction, while its knockdown can suppress hypoxia/reoxygenation (H/R)-induced neuronal cell apoptosis. Such a finding makes BDNF-AS siRNA a useful strategy for treatment of neurodegenerative diseases (Spencer 2008). Also, lncRNA 17A is upregulated in Alzheimer's disease (AD), suggesting that it could directly or indirectly be involved in the mechanism of AD, possibly by impairing the GABA signalling (Massone et al. 2011; Riva et al. 2016). These findings might in part explain the encountered neuronal damage caused by the elevated expression of the studied lncRNAs in epileptic patients.

Remarkably, herein, the upregulation of lncRNAs expression was accompanied by decrements in BDNF, p-CREB, and GABA concentrations along with increments in glutamate and Glutamate/GABA ratio. In vivo*,* and *In vitro* studies have demonstrated that lncRNA BDNF-AS might adversely influence BDNF, where the over-expression of lncRNA BDNF-AS inhibits the expression of BDNF and decreases its protein levels, leading to reduced neuronal growth and synaptic plasticity (Riva et al. 2016). Indeed, the negative correlation between lncRNA BDNF-AS and BDNF, found in the present study, verified the role played by this lncRNA in BDNF post-transcriptional regulation, which may be related to the double-stranded duplexes formed in-between. Interestingly, endothelial dysfunction and blood–brain barrier (BBB) leakage induced by epileptic seizures may be the reason of decreased BDNF levels (Cudna et al. 2017). Low levels of BDNF might in turn aid in the exacerbation of epileptogenesis, especially in focal symptomatic group, as most of the cases suffered from gross anatomical abnormality (Zhong et al. 2017).

Additionally, this study was the first to find a negative correlation between lncRNA 17A and BDNF. Alternatively, a significant positive correlation between BDNF and p-CREB was detected. To our knowledge, BDNF usually increases in the early postnatal period during development and continues to be expressed at high levels in adulthood, being regulated by a variety of molecules including CREB (Dong et al. 2018). It was shown that the promoter region of BDNF contains CRE where p-CREB could bind and promote BDNF transcription as one of CREB target genes (Tao et al. 1998). Mutual interactions between BDNF and CREB presented a positive feedback loop in which BDNF activates CREB transcription, whereas BDNF transcription is activated by its CRE binding to CREB. Interestingly, it has been demonstrated that the activation of CREB/BDNF signals might promote neuronal survival and growth and vice versa in pentylenetetrazole-kindled rats (Yu et al. 2019), which could in part explain the observed decrease in both of them.

An imbalance between excitatory and inhibitory neurotransmission posited a long-standing theorised mechanism during epileptogenesis (Engelborghs et al. 2000). This imbalance was caused by an increase in extracellular glutamate and/or a decrease in GABA concentrations in the brain, resulting in excitotoxicity, seizures and cell death (Bromfield et al. 2006; Guerriero et al. 2015; Bozzi et al. 2018, Sarlo and Holton 2021). Several potential reasons for changes in glutamate concentration have been documented in epilepsy, including glutamate receptor malfunction, glutamate transporter dysfunction and inflammation, all of which supported the study of glutamate concentration beyond focal structural epilepsies (Treiman 2001; López-Pérez et al. 2010; Coulter and Eid 2012; Devinsky et al. 2013; Barker-Haliski and White 2015; Albrecht and Zielińska 2017; Bozzi et al. 2018; Eid et al. 2019; Hanada 2020). On the other side, GABA receptors activate the PI3K/AKT pathway, which in turn protects neurons against stress-induced apoptosis (Tu et al. 2010). Therefore, the decreased GABA as well as the significant elevation in glutamate to GABA ratio, observed in epileptic groups versus healthy control and in symptomatic patients versus idiopathic ones, pointed to the faced damage in those patients and coincided with the afore-mentioned hypothesis that underlines the role of disturbances in the balance between excitatory and inhibitory transmissions, as a major factor leading to epileptogenesis (Morimoto et al. 2004). Another remarkable finding was the negative correlation between lncRNA BDNF-AS and GABA. Since both lncRNA BDNF-AS and GABA affected neuronal apoptosis via PI3K/AKT pathway, we could sepculate that an indirect relationship may coexist, which requires further investigation. Nevertheless, the negative correlation between lncRNA 17A and GABA was previously clarified by Massone et al. (2011) who found that lncRNA17A inhibited the GABA signalling pathway by decreasing GABA_B_ R2 transcription in cerebral cortex of AD. Additionally, the authors reported that the over-expression of lncRNA17A in differentiated human cell lines (SH-SY5Y) produces non-functional GABA_B_ R2 receptors, thereby impairing GABA_B_ mediated signalling. On the other hand, the positive correlations between BDNF, p-CREB and GABA levels with each other could be explained on the basis that GABA influences BDNF by facilitating its expression and increasing its synthesis (Berninger et al. 1995). In the meantime, BDNF promotes synaptic release of GABA via the mitogen-activated protein kinase (MAPK) cascade by increasing Ca^2+^ -induced cAMP accumulation that triggers protein kinase A- dependent CREB phosphorylation and promoting further BDNF expression (Obrietan et al. 2002). Therefore, low GABA levels in this study were expected to lower p-CREB and consequently BDNF expression.

Importantly, data of ROC analysis of the selected lncRNAs and neuromodulators posited that most of them may serve as potential biomarkers for discriminating the symptomatic focal group from idiopathic generalized one, with the highest efficiency obtained by P-CREB, GABA, BDNF-AS, BDNF as well as A17 whereas glutamate was the least efficient. On conducting univariate logistic regression analysis for predicting symptomatic focal epilepsy, most of them are significant predictors with superiority of p-CREB as robust biomarkers for symptomatic focal one.

The main study limitations are small sample size and ethnic issue since this study was conducted at two centres in Cairo which might limit the applications of our findings to wider population. Therefore, further large multi-centred studies are recommended to obtain more data and comparable outcomes.

## Conclusion

During epileptogenesis, increased expression of lncRNAs; BDNF-AS and 17 A was associated with decreased concentrations of p-CREB, BDNF and GABA, together with increased Glutamate/ GABA ratio. The current data suggest a positive feedback loop between GABA/p-CREB/ BDNF and support the role of the studied lncRNAs in proper diagnosis of epileptic patients particularly those with idiopathic generalized from symptomatic focal ones.

## Data Availability

The datasets generated and analysed during the current study are available from the corresponding author on reasonable request.

## References

[CR1] Akyuz E, Polat K, Ates S, Unalmis D, Tokpinar A, Yilmaz S, Kaymak E, Doganyigit Z, Villa C (2020). Investigating cardiac morphological alterations in a pentylenetetrazol-kindling model of epilepsy. Diagnostics.

[CR2] Albrecht J, Zielińska M (2017). Mechanisms of excessive extracellular glutamate accumulation in temporal lobe epilepsy. Neurochem Res.

[CR3] Barker-Haliski M, White HS (2015) Glutamatergic mechanisms associated with seizures and epilepsy. Cold Spring Harbor Perspect Med 5(8):a022863. 10.1101/cshperspect.a022863 10.1101/cshperspect.a022863 PMC4526718 26101204

[CR4] Berg AT, Berkovic SF, Brodie MJ, Buchhalter J, Cross JH, van Emde Boas W, Engel J, French J, Glauser TA, Mathern GW (2010). Revised terminology and concepts for organization of seizures and epilepsies: report of the ILAE Commission on Classification and Terminology, 2005–2009. Wiley Online Library.

[CR5] Berninger B, Marty S, Zafra F, da Penha Berzaghi M, Thoenen H, Lindholm D (1995). GABAergic stimulation switches from enhancing to repressing BDNF expression in rat hippocampal neurons during maturation *In vitro*. Development.

[CR6] Blauwblomme T, Jiruska P, Huberfeld G (2014). Mechanisms of ictogenesis. Int Rev Neurobiol.

[CR7] Bozzi Y, Provenzano G, Casarosa S (2018). Neurobiological bases of autism–epilepsy comorbidity: a focus on excitation/inhibition imbalance. Eur J Neurosci.

[CR8] Bromfield EB, Cavazos JE, Sirven JI (2006) Basic mechanisms underlying seizures and epilepsy. An Introduction to Epilepsy [Internet], American Epilepsy Society, from https://www.ncbi.nlm.nih.gov/books/NBK2510/

[CR9] Coulter DA, Eid T (2012). Astrocytic regulation of glutamate homeostasis in epilepsy. Glia.

[CR10] Crompton DE, Berkovic SF (2009). The borderland of epilepsy: clinical and molecular features of phenomena that mimic epileptic seizures. The Lancet Neurol.

[CR11] Cudna A, Jopowicz A, Mierzejewski P, Kurkowska-Jastrzębska I (2017). Serum metalloproteinase 9 levels increase after generalized tonic-clonic seizures. Epilepsy Res.

[CR12] Czuczwar SJ, Patsalos PN (2001). The new generation of GABA enhancers. CNS Drugs.

[CR13] Devinsky O, Vezzani A, Najjar S, De Lanerolle NC, Rogawski MA (2013). Glia and epilepsy: excitability and inflammation. Trends Neurosci.

[CR14] Devinsky O (2007) Epilepsy: a patient and family guide, Demos Medical publishing, from epilepsy: a patient and family guide - Orrin Devinsky, MD - Google

[CR15] Dong Y, Pu K, Duan W, Chen H, Chen L, Wang Y (2018). Involvement of Akt/CREB signaling pathways in the protective effect of EPA against interleukin-1β-induced cytotoxicity and BDNF down-regulation in cultured rat hippocampal neurons. BMC Neurosci.

[CR16] Eid T, Lee TSW, Patrylo P, Zaveri HP (2019). Astrocytes and glutamine synthetase in epileptogenesis. J Neurosci Res.

[CR17] Engelborghs S, D’hooge R, De Deyn P (2000) Pathophysiology of epilepsy. Acta Neurol Belgica 100(4):201–213. from Pathophysiology of epilepsy - PubMed (nih.gov) 11233674

[CR18] Gómez-Palacio-Schjetnan A, Escobar ML (2013) Neurotrophins and synaptic plasticity. Neurogenesis Neural Plasticity 117–136. 10.1007/7854_2012_231 10.1007/7854_2012_231 23519767

[CR19] Guerriero RM, Giza CC, Rotenberg A (2015) Glutamate and GABA imbalance following traumatic brain injury. Curr Neurol Neurosci Rep 15(5):27. 10.1007/s11910-015-0545-1 10.1007/s11910-015-0545-1 PMC4640931 25796572

[CR20] Hanada T (2020). Ionotropic glutamate receptors in epilepsy: a review focusing on AMPA and NMDA receptors. Biomolecules.

[CR21] Hashemian F, Ghafouri-Fard S, Arsang-Jang S, Mirzajani S, Fallah H, MehvariHabibabadi J, Sayad A, Taheri M (2019). Epilepsy is associated with dysregulation of long non-coding RNAs in the peripheral blood. Front Mol Biosci.

[CR22] Henshall D (2007). Apoptosis signalling pathways in seizure-induced neuronal death and epilepsy. Biochem Soc Transac.

[CR23] Henshall DC, Kobow K (2015). Epigenetics and epilepsy. Cold Spring Harb Perspect Med.

[CR24] Hewson C, Capraro D, Burdach J, Whitaker N, Morris KV (2016). Extracellular vesicle associated long non-coding RNAs functionally enhance cell viability. Non-coding RNA Res.

[CR25] Hu Y, Lund IV, Gravielle MC, Farb DH, Brooks-Kayal AR, Russek SJ (2008). Surface expression of GABAA receptors is transcriptionally controlled by the interplay of cAMP-response element-binding protein and its binding partner inducible cAMP early repressor. J Biol Chem.

[CR26] Jagasia R, Steib K, Englberger E, Herold S, Faus-Kessler T, Saxe M, Gage FH, Song H, Lie DC (2009). GABA-cAMP response element-binding protein signaling regulates maturation and survival of newly generated neurons in the adult hippocampus. J Neurosci.

[CR27] Jang Y, Moon J, Lee S-T, Jun J-S, Kim T-J, Lim J-A, Park B-S, Yu J-S, Park D-K, Yang AR (2018). Dysregulated long non-coding RNAs in the temporal lobe epilepsy mouse model. Seizure.

[CR28] Landmark CJ (2008). Antiepileptic drugs in non-epilepsy disorders. CNS drugs.

[CR29] Lasoń W, Chlebicka M, Rejdak K (2013). Research advances in basic mechanisms of seizures and antiepileptic drug action. Pharmacol Rep.

[CR30] Leite JP, Neder L, Arisi GM, Carlotti CG, Assirati JA, Moreira JE (2005). Plasticity, synaptic strength, and epilepsy: what can we learn from ultrastructural data?. Epilepsia.

[CR31] Lerche H, Shah M, Beck H, Noebels J, Johnston D, Vincent A (2013). Ion channels in genetic and acquired forms of epilepsy. J Physiol.

[CR32] Lipovich L, Dachet F, Cai J, Bagla S, Balan K, Jia H, Loeb JA (2012). Activity-dependent human brain coding/noncoding gene regulatory networks. Genetics.

[CR33] López-Pérez SJ, Ureña-Guerrero ME, Morales-Villagrán A (2010). Monosodium glutamate neonatal treatment as a seizure and excitotoxic model. Brain Res.

[CR34] Marler KJ, Suetterlin P, Dopplapudi A, Rubikaite A, Adnan J, Maiorano NA, Lowe AS, Thompson ID, Pathania M, Bordey A (2014). BDNF promotes axon branching of retinal ganglion cells via miRNA-132 and p250GAP. J Neurosci.

[CR35] Massone S, Vassallo I, Fiorino G, Castelnuovo M, Barbieri F, Borghi R, Tabaton M, Robello M, Gatta E, Russo C (2011). 17A, a novel non-coding RNA, regulates GABA B alternative splicing and signaling in response to inflammatory stimuli and in Alzheimer disease. Neurobiol Dis.

[CR36] Mohammadi A, Amooeian VG, Rashidi E (2018) Dysfunction in brain-derived neurotrophic factor signaling pathway and susceptibility to schizophrenia, Parkinson’s and Alzheimer’s diseases. Curr Gene Ther 18(1):45–63. 10.2174/1566523218666180302163029 10.2174/1566523218666180302163029 29512462

[CR37] Morimoto K, Fahnestock M, Racine RJ (2004). Kindling and status epilepticus models of epilepsy: rewiring the brain. Progress Neurobiol.

[CR38] Obrietan K, Gao X-B, Van Den Pol AN (2002). Excitatory actions of GABA increase BDNF expression via a MAPK-CREB–dependent mechanism—a positive feedback circuit in developing neurons. J Neurophysiol.

[CR39] Pákozdy Á, Leschnik M, Tichy A, Thalhammer J (2008). Retrospective clinical comparison of idiopathic versus symptomatic epilepsy in 240 dogs with seizures. Acta Vet Hungarica.

[CR40] Riva P, Ratti A, Venturin M (2016). The long non-coding RNAs in neurodegenerative diseases: novel mechanisms of pathogenesis. Curr Alzheimer Res.

[CR41] Sarlo GL, Holton KF (2021). Brain concentrations of glutamate and GABA in human epilepsy: a review. Seizure.

[CR42] Shao Y, Chen Y (2017). Pathophysiology and clinical utility of non-coding RNAs in epilepsy. Front Mol Neurosci.

[CR43] Shorvon SD (2011) The etiologic classification of epilepsy. Epilepsia 52(6):1052–1057. 10.1111/j.1528-1167.2011.03041.x 10.1111/j.1528-1167.2011.03041.x 21449936

[CR44] Sirven JI (2015) Epilepsy: a spectrum disorder. Cold Spring Harbor Perspect Med 5(9): a022848, from Epilepsy: A Spectrum Disorder (cshlp.org) 10.1101/cshperspect.a022848 PMC4561391 26328931

[CR45] Sopic D, Aminifar A, Atienza D (2018) e-glass: A wearable system for real-time detection of epileptic seizures. 2018 IEEE International Symposium on Circuits and Systems (ISCAS), IEEE

[CR46] Spencer JP (2008). Flavonoids: modulators of brain function?. Br J Nutr.

[CR47] Tao X, Finkbeiner S, Arnold DB, Shaywitz AJ, Greenberg ME (1998). Ca2+ influx regulates BDNF transcription by a CREB family transcription factor-dependent mechanism. Neuron.

[CR48] Treiman DM (2001). GABAergic mechanisms in epilepsy. Epilepsia.

[CR49] Tu H, Xu C, Zhang W, Liu Q, Rondard P, Pin J-P, Liu J (2010). GABAB receptor activation protects neurons from apoptosis via IGF-1 receptor transactivation. J Neurosci.

[CR50] Vezzani A, French J, Bartfai T, Baram TZ (2011). The role of inflammation in epilepsy. Nat Rev Neurol.

[CR51] Villa C, Lavitrano M, Combi R (2019). Long non-coding RNAs and related molecular pathways in the pathogenesis of epilepsy. Int J Mol Sci.

[CR52] Wang G, Zhu Z, Xu D, Sun L (2020). Advances in understanding CREB signaling-mediated regulation of the pathogenesis and progression of epilepsy. Clin Neurol Neurosurg.

[CR53] Wang T (2021) Epilepsy, headache and pain associated with neurological disorders. Acupuncture for Brain, Springer, pp 191–203. 10.1007/978-3-030-54666-3_14

[CR54] Yamada K, Mizuno M, Nabeshima T (2002). Role for brain-derived neurotrophic factor in learning and memory. Life Sci.

[CR55] Yu X, Guan Q, Wang Y, Shen H, Zhai L, Lu X, Jin Y (2019). Anticonvulsant and anti-apoptosis effects of salvianolic acid B on pentylenetetrazole-kindled rats via AKT/CREB/BDNF signaling. Epilepsy Res.

[CR56] Zhang H, Liu C, Yan T, Wang J, Liang W (2018). Long noncoding RNA BDNF-AS is downregulated in cervical cancer and has anti-cancer functions by negatively associating with BDNF. Arch Biochem Biophys.

[CR57] Zhi H, Lian J (2019). LncRNA BDNF-AS suppresses colorectal cancer cell proliferation and migration by epigenetically repressing GSK-3β expression. Cell Biochem funct.

[CR58] Zhong J-B, Li X, Zhong S-M, Liu J-D, Chen C-B, Wu X-Y (2017). Knockdown of long noncoding antisense RNA brain-derived neurotrophic factor attenuates hypoxia/reoxygenation-induced nerve cell apoptosis through the BDNF–TrkB–PI3K/Akt signaling pathway. Neuroreport.

